# Novel molecular, cytotoxical, and immunological study on promising and selective anticancer activity of Mung bean sprouts

**DOI:** 10.1186/1472-6882-12-208

**Published:** 2012-11-05

**Authors:** Rand R Hafidh, Ahmed S Abdulamir, Fatimah Abu Bakar, Farid Azizi Jalilian, Faridah Abas, Zamberi Sekawi

**Affiliations:** 1 Department of Microbiology, College of Medicine, Baghdad University, Baghdad, Iraq; 2 Institute of Bioscience, Universiti Putra Malaysia, 43400 UPM Serdang, Selangor Darul Ehsan, Malaysia; 3 Department of Microbiology, College of Medicine, Al-Nahrain University, Baghdad, Iraq; 4 Department of Medical Microbiology and Parasitology, Faculty of Medicine and Health Sciences, Universiti Putra Malaysia, 43400 UPM Serdang, Selangor Darul Ehsan, Malaysia; 5 Clinical Microbiology Research Center, Ilam University of Medical Sciences, Ilam, Iran; 6 Department of Food Science, Faculty of Food Science and Technology and Institute of Bioscience, Universiti Putra Malaysia, 43400 UPM Serdang, Selangor Darul Ehsan, Malaysia

## Abstract

**Background:**

The anticancer and immunomodulatory activity of mung bean sprouts (MBS) and the underlying mechanisms against human cervical and hepatocarcinoma cancer cells were explored.

**Methods:**

MBS cytotoxicity and MBS-induced anticancer cytokines, TNF-α and IFN-β from cancer cells, and immunological cytokines, IL-4, IFN-γ, and IL-10 from peripheral mononuclear cells (PMNC) were assessed by MTS and ELISA assays. Apoptotic cells were investigated by flow cytometry. The expression level of apoptotic genes (Bax, BCL-2, Capsases 7–9) and cell cycle regulatory genes (cyclin D, E, and A) and tumor suppressor proteins (p27, p21, and p53) was assessed by real-time qPCR in the cancer cells treated with extract IC50.

**Results:**

The cytotoxicity on normal human cells was significantly different from HeLa and HepG2 cells, 163.97 ± 5.73, 13.3 ± 0.89, and 14.04 ± 1.5 mg/ml, respectively. The selectivity index (SI) was 12.44 ± 0.83 for HeLa and 11.94 ± 1.2 for HepG2 cells. Increased levels of TNF-α and IFN-β were observed in the treated HeLa and HepG2 culture supernatants when compared with untreated cells. MBS extract was shown to be an immunopolarizing agent by inducing IFNγ and inhibiting IL-4 production by PBMC; this leads to triggering of CMI and cellular cytotoxicity. The extract induced apoptosis, in a dose and time dependent manner, in treated HeLa and HepG2, but not in untreated, cells (P < 0.05). The treatment significantly induced cell cycle arrest in G0/G1 in HeLa cells. The percentage of cells in G0/G1 phase of the treated HeLa cells increased from 62.87 ± 2.1%, in untreated cells, to 80.48 ± 2.97%. Interestingly, MBS IC50 induced the expression of apoptosis and tumor suppressor related genes in both HeLa and HepG2 cells. MBS extract succeeded in inducing cdk-inhibitors, p21, p53, and p27 in HeLa cells while it induced only p53 in HepG2 cells (P < 0.05). This is a clue for the cell type- specific interaction of the studied extract. These proteins inhibit the cyclin-cdk complexes apart from the presence of some other components that might stimulate some cyclins such as cyclin E, A, and D.

**Conclusion:**

MBS extract was shown to be a potent anticancer agent granting new prospects of anticancer therapy using natural products.

## Background

More attention has recently been given to the role of plant-derived compounds as promising nutraceuticals for controlling various disorders such as cardiovascular, neurological, neoplastic and immunological diseases [1]. The phytochemical compounds are found to be integral components of human diet. They are commonly present as constituents of flowering plants, particularly of food plants [2].

Studies verified that phytochemicals are able to alter the likelihood of carcinogenesis in every stage of cancer process in a way reducing the risk but usually in a favorable direction [3]. Interestingly, the main activity of these compounds depends on the fact that the exposure of human cells to a wide variety of chemoprotective compounds confers resistance against a broad set of carcinogens [4]. Much information exists nowadays on the antitumor action of plants, and many in vitro studies have concentrated on the direct and indirect actions of phytochemicals on tumor cells, and have found a variety of anticancer effects such as cell growth [5], kinase activity inhibition [6], apoptosis induction [7], and suppression of the secretion of matrix metalloproteinases, and tumor invasive behavior [8]. Furthermore, some studies reported the impairment of *in vivo* angiogenesis by dietary phytochemicals [9]. Therefore, the discovery of new anticancer agents from plant-derived substances is considered to be a highly promising approach in order to enrich the pharmaceutical field with effective drugs of lower side effects.

Besides, plants produce a vast number of natural products which have antimicrobial and immunomodulating potential as defense mechanisms for adapting to various environmental insults [10]. Many natural compounds have shown a significant ability to regulate immune responses [11]. Some of these phytochemicals with immunomodulating effects are isoflavonoids, indoles, phytosterols, polysaccharides, sesquiterpenes, alkaloids, glucans, tannins, a variety of vitamins and trace minerals that function as antioxidants and co-enzymes, and many other phytochemical substances [12].

It is clear that human immune response is a highly complex and extraordinarily sophisticated system involving both innate and adaptive mechanisms [13]. Immunomodulating activity refers to biological or pharmacological effects of compounds on humoral or cellular aspects of the immune response. In other words, immunomodulation is the process of modifying an immune response in a positive or negative manner by administration of a drug or a compound [11]. Although the field of study of immune enhancing compounds is relatively not new [14], natural products from plants represent a rich and promising source of novel molecules with immunomodulating properties that may augment a disease recovery alone or together with commercially known drugs.

For the first time in the field, the current study investigated the in vitro selective cytotoxic and immunomodulatory effects of mung bean sprout (*Vigna radiata* L.) methanol crude extracts on human cancer and peripheral mononuclear cells (PMNC), respectively. The rationale behind testing the anticancer and immunomodulatory effects of MBS extract is that first, MBS has not been assessed as anticancer or as immunomodulatory agent before, second, MBS is a germinating plant which usually possesses very high levels of antioxidants that are well known to act as potent anticancer and immunomodulatory agents. More in depth, this study explored thoroughly the underlying mechanisms of the newly discovered findings in the current study, namely, the novel anticancer and immunomodulatory effects of mung bean sprout (*Vigna radiata* L.) methanol extract by using cytological and molecular assays for assessing and measuring anticancer cytokines, cell cycle phases, percentage of apoptotic cells, expression level of cell cycle genes, tumor suppressor genes and apoptosis genes in MBS treated and untreated cancer cells. Moreover, PBMC-secreted cytokines, in response MBS extract exposure, were measured as an indicator for the immunomodulatory effect of MBS. Therefore, the ultimate goal of the current study is to find a natural anticancer product able to inhibit the proliferation of human cancer cell lines with high selectivity index, safe usage, and effective anticancer activity. This study has been patented in Malaysian intellectual property (MyIPO) under Malaysian patent application number PI2011001617 on 12th April 2011 (http://www.rmc.upm.edu.my/upmip/index.php?content=getfaculty&ipid=683&ipdetailid=671&projectlead=151&cluster=3&fac=8) and the current patented research has been considered for commercialization by University Putra Malaysia.

## Methods

### Ethical approach

The current study was conducted in compliance with Helsinki Declaration for ethical approaches of conducting scientific research. This study was approved by the ethical committee of University Putra Malaysia in Kuala Lumpur.

### Preparation of the MBS extract

Fresh mung bean sprouts (MBS), devoid of any preservative antimicrobials was purchased from local markets in the State of Selangor, Malaysia. The growth of mung beans and the germination of the sprouts were done in Selangor State. The mung bean sprouts were left to dry in dark area for three days at room temperature 23-25°C. After the dryness of sprouts, they were ground to powder. The ground powder was extracted 1:10 wt/v with 2.4 M HCl acidified methanol (Merck, Darmstadt, Germany) to extract all, free and conjugated, components of phenolic compounds [15]; the ground powder was then soaked in dark area for three days at room temperature. The supernatant was collected after filtration and the fresh solvent was added to the plant material. The extraction procedure was repeated twice and the collected extracts were evaporated to dryness under vacuum at 40°C by using rotary evaporator. To remove the effect of the acidity from the crude extract, the crude extract of MBS was neutralized to exclude any pH-related effect. The pH for MBS extract was neutral ranging from 6.8 to 7.0. The dried extracts were stored at −18°C in a desiccant until further use.

### Preparation of stock extract

The stock extract for MBS was prepared by redissolving the MBS extract powder in dimethyl sulfoxide (DMSO) 0.1%, (BIO BASIC INC., NY, USA). This concentration is usually non toxic to cell culture [16]. Moreover, this concentration was tested in the current study and was found to be non toxic to HeLa and HepG2 cells. The vitality of cells incubated with DMSO (0.1%) was tested by MTT assay for different time intervals and was compared with that of control cells We found that both tested HeLa and HepG2 cells kept their vitality and freshness with DMSO concentration of 0.1% when compared to control cells with fresh medium [data not shown]. Afterwards, the dissolved suspension was centrifuged at 134 g for 10 min and filtrated by 0.22 μm Millipore filters (Nalgene, UK). The stock was stored at −20°C until it further use. The concentration of the stock extract was determined as required.

### Cancer cells propagation and cryopreservation

Two human cancer cell lines, namely, cervix adenocarcinoma cells (HeLa; ATCC CCL-2) and hepatocellular carcenoma (HepG2; ATCC HB-8065), were used to evaluate the cytotoxic effects of MBS extract. Cells were propagated as monolayer under humidified 5% CO_2_ atmosphere at 37°C in Roswell park memorial institute-1640 (RPMI-1640) culture medium w/L-glutamine (biowest, Florida, USA) supplemented with 10% fetal bovine serum (FBS) (Sigma, Germany), 50 U/ml penicillin-streptomycin (biowest, Florida, USA), and 2.5 μg/ml amphotericin B (biowest, Florida, USA). Part of the cells was cryopreserved for future work in liquid nitrogen (−196°C) after suspending in RPMI-1640 cryospreserved medium supplemented with 10% FBS, 20% DMSO, 50 U/ml penicillin-streptomycin, and 2.5 μg/ml amphotericin B.

### Isolation of human peripheral blood mononuclear cells (PBMC)

Human PBMC were isolated by density gradient centrifugation technique from heparinized whole blood. Thirty milliliters of fresh heparinized blood sample isolated from normal donor and diluted in phosphate buffer saline (PBS), (CALBIOCHEM, Darmstadt, Germany) were gently laid over 15 ml of Ficoll Hypaque (GE Healthcare Bio-sciences AB, Sweden) and were spun at 500 g for 20 min at 25°C. PBMC were collected from the interface of spun blood samples and were washed three times with PBS by centrifugation at 500 g for 10 min at 4°C. The supernatant was discarded and the cells were suspended in RPMI-1640 culture medium. Trypan blue solution 0.4% (Sigma, Germany) was used to count the cells into an appropriate concentration and the viability of cells was checked; the required range of cells’ viability is 95-99%.

### MTS tetrazolium assay

In order to determine the viable cells in proliferation or cytotoxicity assays, the MTS colorimetric method (the Cell Titer 96® Aqueous One Solution Cell Proliferation Assay, Promega, USA) was used. MTS [3-(4,5-dimethylthiazol-2-yl)-5-(3-carboxymethoxyphenyl)-2(4-sulfophenyl)-2H-tetrazolium] is reduced by dehydrogenase enzymes in metabolically active cells producing soluble colored formazan in tissue culture medium. The quantity of formazan products, measured at 490 nm absorbance after 4h incubation time, is directly proportional to the number of living cells in the culture [17]. The absorbance was measured using a 96-well plate ELISA reader (Sunrise Basic Tecan, Grödig, Austria).

### Proliferation and cytotoxicity assays for PBMC

Purified mononuclear cells, 2 × 10^5^ cell/well, were cultured in quadruplicate in 96-well U-bottom tissue culture plates (Orange Scientific, Europe) with 2-fold serial dilutions of each extract in RPMI-1640 culture medium to a final volume of 200 μl/well. The MBS extract concentrations ranged from 300 to 9.37 mg/ml. The negative control wells, in quadruplicates, contained PBMC in RPMI-1640 culture medium (200 μl/well). The positive control wells, in quadruplicates, contained PBMC with Concavalin A (Con A), (10 μg/ml), a T-cell specific mitogen (Sigma, Germany), at a final volume of 200 μl/well. The plates were used to assess the cytotoxicity or the stimulatory effect of the extracts on PBMC proliferation. Cultures were incubated in humidified 5% CO_2_ atmosphere for 72h at 37°C. After 72h, 20 μl of MTS solution with 100 μl of RPMI-1640 culture medium were added to each well. The plates were incubated for four hours at the same conditions. Later, the absorbance was measured at 490 nm (reference 690 nm) using a 96-well plate ELISA reader. Each experiment was repeated for three times with four wells per dilution in each run.

The extract effect to stimulate PBMC proliferation was calculated using the following formula:

$$\text{Proliferation}\;\%=\left[\left(\text{ODt}/\text{ODc}\right)–1\right]\times 100$$

Where (ODt) indicates the optical density of the tested extract and (ODc) indicates the optical density of the negative control [18].

On the other hand, the data of the extract cytotoxicity against PBMC was calculated using the following formula:

$$\text{Cytotoxicity}\;\%=\left[1–\left(\text{ODt}/\text{ODc}\right)\right]\times 100$$

Where (ODt) indicates the optical density of the tested extract and (ODc) indicates the optical density of the negative control. Accordingly, the concentration of 50% inhibition (CC50) was the concentration that achieved 50% cytotoxicity against PBMC [19].

### Cytotoxicity on human cancer cell lines

Cytotoxicity assay was performed according to the established method of Mena-Rejon et al. (2009), where 1 ×k 10^5^ cell/well viable HeLa and HepG2 cells were grown in RPMI-1640 culture medium in 96-well flat-bottom tissue culture plates (Orange Scientific, Europe). The plates were incubated in humidified 5% CO_2_ for 24h at 37°C. When cells reached > 80% confluence, the medium was replaced with 200 μl/well of 2-fold serial dilutions of MBS extract from 164 to 10.25 mg/ml prepared in RPMI-1640 maintenance medium (with 2% FBS). The concentration of MBS extract was 328 mg/ml. The negative control wells contained DMSO (0.1%) in RPMI-1640 maintenance medium with final volume of 200 μl/well. All the plates were incubated in humidified 5% CO_2_ for 24h at 37°C. Later, all the wells’ contents were removed and replaced with 200 μl/well of RPMI-1640 maintenance medium. The plates were re-incubated for 48 h at the same conditions. Afterwards, 20 μl of MTS solution with 100 μl of RPMI-1640 culture medium were added to each well. The plates were incubated for four hours in the same conditions. The absorbance was measured at 490 nm (reference 690 nm) using a 96-well plate ELISA reader. Each experiment was repeated for three times with four wells per dilution in each run [20].

The concentration of the extract that killed 50% of the cells (IC50) was calculated using the following formula:

$$\text{Cytotoxicity}\;\%=\left[1–\left(\text{ODt}/\text{ODc}\right)\right]\times 100$$

Where (ODt) indicates the optical density of the tested extract and (ODc) indicates the optical density of the negative control. The selectivity index (SI) was the ratio of CC50 (cytotoxicity on PBMC) to the IC50 (cytotoxicity on human cancer cells), [19].

### Cytokine production by PBMC

In order to evaluate the immunomodulatory effect of MBS extract, the cytokine level produced from PBMC after treatment with MBS extract was investigated. PBMC at 2×10^5^ cell/well were cultured, in triplicates, in 96-well U-bottom tissue culture plates with 2-fold serial dilutions of the extract in RPMI-1640 culture medium to a final volume of 200 μl/well. The extract concentrations ranged from 100 to 3.12 mg/ml. The negative control wells, in triplicates, contained 200 μl/well of PBMC in RPMI-1640 culture medium. After 24 h of incubation at 37°C in humidified 5% CO_2_ atmosphere, the plates were centrifuged at 300 g for 10 min. The supernatant was collected and centrifuged at 1000 g for 10 min to be ready to determine the cytokine level produced into the medium [21].

### Cytokine production by human cancer cell lines

The level of anticancer cytokines, IFN-β and TNF-α, was investigated in treated and untreated HeLa and HepG2 cells. HeLa and HepG2 cells at 1 × 10^5^ cell/well were grown in RPMI-1640 culture medium in 96-well flat-bottom tissue culture plates in a humidified 5% CO_2_ atmosphere for 24 h at 37°C. Later, serial 2-fold dilutions of MBS 10 to 0.31 mg/ml prepared in RPMI-1640 maintenance medium were added to the cells, in triplicates, to a final volume of 200 μl/well. The MBS extract stock concentration was 20 mg/ml. The negative control wells, in triplicates, contained cells with RPMI-1640 maintenance medium only with final volume of 200 μl/well. The plates were incubated for 48h at the same conditions. Afterwards the supernatants from all the wells were removed and centrifuged at 1000 g for 10min to be ready for the ELISA technique [22].

### Measuerment of cytokines produced by PBMC and cancer cells

The levels of immunomodulatory cytokines, IL-2, IL-4 and IFN-γ, in the PBMC culture supernatant with and without extract treatment, and the levels of anticancer cytokines, IFN-β and TNF-α, in the supernatant of cultured treated and non-treated cancer cells, were measured. The procedures pursued were according to the manufacturers’ instructions of each kit. For IL-2 and IL-4, the used kit was human enzyme immunometric assay (EIA) kits (Cayman, USA), for IFN-γ, a human interferon gamma ELISA kit (abcam, USA) was used, and for anticancer cytokines, human IFN-β ELISA (abcam, USA) and TNF-α human enzyme immunometric assay (EIA) kits (Cayman, USA) were used.

Each microtiter plate was already coated with monoclonal antibodies specific for the corresponding cytokine. The standards of IL-2 and IL-4 were reconstituted in the same matrix of the samples, namely RPMI-1640 medium plus each 2-fold serial dilution of the extract; this step is needed to match the color effect of unpurified samples that might persist even after the subsequent washing steps. Accordingly, a standard curve was made for each dilution of the extracts used. The IL-2 or IL-4 standards were prepared as 2-fold serial dilutions ranging from 250 to 3.9 pg/ml in eight tubes of 15 ml capacity (Orange Scientific, Europe); the eighth tube contained zero concentration of IL-2 or IL-4 standard.

For IFN-γ, standards were freshly prepared by reconstituting in the standard diluents buffer to give a stock concentration of 400 pg/ml. Two hundred microliter of this stock was added, in triplicates, to the already coated microtiter plates provided by the kit. Each plate was coated with monoclonal IFN-γ specific antibodies. From stock wells, serial 2-fold dilutions of IFN-γ standard were prepared by diluting the standard in a matrix similar to that of the samples which is RPMI-1640 culture medium plus each dilution of the extracts used. This step is needed to match the color effect of unpurified samples that might persist even after the subsequent washing steps. Accordingly, a standard curve was made for each dilution of the extracts used. IFN-γ standard concentrations ranged from 400 to 12.5 pg/ml; and the last standard contained zero concentration of IFN-γ standard in the sample matrix.

For immunomodulatory cytokines, IL-2, IL-4, IFN-γ, and anticancer cytokines, IFN-β and TNF-α, one hundred μl of standards, samples, and conjugates were all assayed as triplicates. For IL-2 and IL-4, the absorbance was measured at 412 nm (reference 690 nm) using a 96-well plate ELISA reader. The assay was repeated for three times for each sample. The concentrations of samples’ IL-2 and IL-4 were determined by extrapolating their OD values with that of the generated standard curves. For IFN-γ, the absorbance was measured at 450 nm (reference 620 nm) by ELISA reader. The sample concentrations were determined by extrapolating OD values to IFN-γ concentrations using the generated linear standard curves (the average absorbance on the vertical axis versus the corresponding IFN-γ standard concentration on the horizontal axis). One hundred μl of sample, conjugates and substrate-chromogen were used according to the guidelines of the kits manufacturer. Later, the absorbance was measured at 412 nm (reference 690 nm) and at 450 nm (reference 620 nm) using a 96-well plate ELISA reader for TNF-α and IFN-β plates, respectively. The assay was repeated for three times for each sample. The concentrations of the cytokines were determined by extrapolating their OD values with that of the generated standard curves.

### Analysis of apoptosis

#### Flow cytometry analysis for cell apoptosis

Flow cytometry can rapidly quantify and evaluate the properties of apoptotic cells. It can give information on the ratio of apoptotic cells, based on the cellular size or DNA contents. It is well known that several differences are present between apoptotic cells and normal cells. These differences can be utilized by flow cytometric techniques for apoptosis detection [23]. The cells (HeLa and HepG2) were seeded (1 × 10^5^ cell/well) in 6-wells tissue culture plates and were incubated in a humidified 5% CO_2_ atmosphere for 24 h at 37°C. The medium was then replaced with RPMI-1640 maintenance medium with or without MBS extract and was incubated for further 24 h at the same conditions. The cell treatment was divided into two groups. In the first group (dose-dependent group), the effect of three 2-fold serial dilutions of the extract (concentration > IC50 > concentration) was investigated regarding the level of apoptosis, if any, after a fixed time. The concentrations of MBS extract were 26.6, 13.3, and 6.65 mg/ml with HeLa cells and were 28.08, 14.04, and 7.02 mg/ml with HepG2 cells. In the second group (time-dependent group), the IC50 of MBS extract was used to investigate the level of cells’ apoptosis as well as cell cycle arrest after different time intervals of incubation with the extract. For the first group, after 24 h, the cells were harvested and transferred to 15 ml tubes. All of the tubes were centrifuged at 190 g for 10 min. The supernatants were discarded and the pellets were washed two times by cold PBS. Later, the pellets were resuspended in 70% ice-cold ethanol with PBS, 1:10 v/v, and were incubated for 2h at −20°C. Then, all the supernatants were aspirated after centrifugation at 500 g for 10 min. The washing step by PBS was repeated and the supernatants were aspirated. The pellets were resuspended in 500 μl of DNA staining solution containing 25 μl of propidium iodide (PI) 1 mg/ml (MP Biomedicals, LLC, IIIKrick, France), a double-stranded nucleic acid intercalating agent, and 50 μl Ribonuclease A from bovine pancrease (1 mg/ml), (Sigma, Germany) in PBS. All the tubes were incubated on ice in dark area for 30min [24]. The assay was measured in duplicate for each sample. The propidium iodide fluorescence of individual nuclei was measured using CyAn ADP apparatus (BECKMAN COULTER, USA). The software Summit (V4.3) was used to analyze the flow cytometry results.

For the second group of the flow cytometry analysis, the stock extract was prepared and the same method mentioned earlier for the first group was used except for the following differences: the IC50 for MBS extract with HeLa and HepG2 cells was used to treat the cells. The IC50 of MBS extract was 13.3 mg/ml with HeLa cells and 14.04 mg/ml with HepG2 cells. The group was subdivided into seven treatments (8, 12, 16, 20, 24, 48 and 72 h). The assay was measured in duplicate for each sample in the specific time of treatment.

#### Detection of apoptosis- and cell cycle arrest- related genes by real time quantitative PCR (qRT-PCR)

The current trend of research uses advanced techniques in molecular biology to study the apoptosis- and cell cycle arrest- related genes’ expression such as Bcl-2 family, members of caspase family, tumor suppressor proteins, and cyclins. Nowadays, by using the reverse transcription-polymerase chain reaction (RT-PCR), the scientists can demonstrate the level of mRNA expression of apoptosis and cell cycle proteins even though they are expressed only in a small cell population in tissues [25].

#### RNA extraction

The cells (HeLa and HepG2) were seeded (1 × 10^5^ cell/well) in 6-wells tissue culture plates and were incubated in a humidified 5% CO_2_ atmosphere for 24 h at 37°C. The medium was then replaced with RPMI-1640 maintenance medium either alone or with MBS extract, in duplicates, and the medium was incubated at the same conditions. The IC50 for MBS extract against each type of cells was used. The IC50 of MBS extract was 13.3 mg/ml with HeLa cells and 14.04mg/ml with HepG2 cells. After determining the best timing for studying the apoptosis and cell cycle arrest by flow cytometry, treatment of cells with extract for 12, 16, and 20 h was conducted. At the end of 12, 16, or 20h of extract treatment, the cells were harvested and transferred to 15 ml tubes. After centrifuging the tubes at 134 g for 5 min, the supernatants were discarded. The pellets were resuspended in PBS and were washed for four times.

It is noteworthy to mention that one of the most important steps preceding the synthesis of good quality cDNA is the isolation of intact (undegraded) total RNA from cultured cells or tissues [25]. Total RNA was isolated using GF-1 kit (Vivantis Technologies, Malaysia). According to the manufacturer’s protocol for RNA isolation from cell culture, 1 × 10^7^ cells were precipitated in 1.5 ml microtubes (Eppendrof, Hamberg, Germany) at 1000 × g for 5 min. The cell pellets were resuspended in 700 μl of lyses buffer (Buffer TR) with vigorous mixing by vortexing. This buffer is specially formulated to inactivate cellular RNases together with cell lysis. Later, the lysed cells were transferred to homogenization columns assembled in a collection tubes. The columns were centrifuged at 10.000 × g for 2 min. The flow-though was saved and equal volume of 80% ethanol (700 μl) was added. The lysed cells were mixed thoroughly by pipetting and were transferred into RNA binding columns assembled in collection tubes. RNA binding columns were centrifuged at 10.000 × g for 1 min and the flow-though was discarded. The columns were washed by adding 500 μl of washing buffer and were centrifuged at 14.000 × g for 1 min. The flow-though was discarded and all of the DNA fragments were removed by DNase I treatment. Seventy microlitter of DNase I Digestion Mix were added to RNA binding columns and were incubated at room temperature for 15 min. DNase I Digestion Mix was composed of DNase I (7 μl), digestion buffer (56 μl), and digestion enhancer (7 μl). Then, 500 μl of inhibitor removal buffer were added to the columns which were centrifuged at 14.000 × g for 1 min. The columns were washed with 500 μl washing buffer for two times with centrifugation at 10.000 × g for 1 min for each run. Further centrifugation at 10.000 × g for 1 min was done to remove any traces of buffer. Total RNA was collected by placing the columns into new 1.5 ml microtubes with 60 μl RNase-free water addition and standing for 1 min. The microtubes were centrifuged at 10.000 × g for 1 min. RNA quality and quantity were determined by Life Science UV/Vis Spectrophotometer, DU Series 700 (BECKMAN COULTER, USA).The isolated RNA was stored at −80°C and was ready for use in downstream application, namely, qRT-PCR.

#### Real time quantitative RT-PCR

One microgram of the isolated RNA from each sample was reverse-transcribed by iScript™ cDNA Synthesis Kit (BIO-RAD, Hercules, Canada). According to the manufacturer’s protocol, 4 μl of 5 × iScript reaction mix were mixed with 1 μl iScript reverse transcriptase and 15 μl of RNA template in 1.5 ml microtubes to give final volume of 20 μl per reaction. The complete reaction mix was incubated for 5 min at 25°C then for 30 min at 42°C using Thermo Bath, ALB64 (FINEPCR, Seoul, Korea). The incubation temperature was increased to 85°C for 5 min. Finally, cDNA was stored at −80°C for qRT-PCR reaction.

Real-time quantitative PCR reaction was conducted using SsoFast™ EvaGreen® Supermix (BIO-RAD, Hercules, Canada). Depending on the manufacturer’s protocol, 10 μl of 1x SsoFast EvaGreen supermix were mixed with 7 μl RNase/DNase free water. One microlitter of forward primer (500 nM) and 1 μl of reverse primer (500 nM) were added to the previous mix (Table 1). Finally, 1 μl of cDNA template corresponding to 50 ng of total RNA was added. The PCR reaction (20 μl) was run for 40 cycles using CFX96™ Real-Time System (BIO-RAD, Hercules, Canada). Cycling conditions were 95°C for 3 min, 95°C for 10 sec, 55-61°C for 30 sec, and 72°C for 20 sec. PCR reaction for cDNA templates from untreated HeLa and HepG2 cells were used as negative controls. The PCR reaction was run in triplicate for each target gene. PCR reaction mix without cDNA template was used to detect any contamination. At the end of the amplification, measurement of Eva Green fluorescence was done continuously with the conduction of the melting curve analysis by slow heating at 0.5°Cs^-1^ increments from 70 to 95°C, with continuous fluorescence collection. Accordingly, a melting curve was generated at the end of the PCR amplification for monitoring the specificity of PCR reaction. Melting curve analysis of the negative first derivative was pursued. Beta-actin was used as a housekeeping gene (reference gene) to normalize the mRNA expression of target genes. Because PCR efficiency may vary among different primers, the calculation of PCR primers’ efficiency is essential for obtaining accurate measurements of the relative expression of the mRNA of target genes [26]. For this reason, a standard curve was created by diluting template cDNA of each single primer used in this study. The cDNA template for each primer was serially diluted (10^-1^ to 10^-7^); each dilution serves as a standard. In this reaction, cDNA template was used from samples with high expression to the target of interest. The amplification efficiency can be obtained by analyzing the slope of the log-linear portion of the standard curve. When the logarithm of the initial template concentration is plotted on the x axis and the threshold cycle (C_t_) is plotted on the y axis, PCR efficiency is calculated according to the following equation [26]:

$$\text{PCR efficiency}=10^{-1/\text{slope}}–1$$

**Table 1:** **Primers used in Real**-**Time quantitative PCR analysis** (**Vivantis Technologies**, **Malaysia**)

	**Forward primer**	**Reverse primer**
Bax	CAC CAG CTC TGA GCA GAT	GCG AGG CGG TGA GCA CTC
BCL-2	TAC CTG AAC CGG CAC CTG	GCC GTA CAG TTC CAC AAA GG
Caspase 7	GTC TCA CCT ATC CTG CCC TCA	TTC TTC TTC TGC CTC ACT GTC
Caspase 8	GAA AAG CAA ACC TCG GGG ATA C	CCA AGT GTG TTC CAT TCC TGT C
Caspase 9	CCA GAG ATT CGC AAA CCA GAG G	GAG CAC CGA CAT CAC CAA ATC C
Cyclin D	AGA CCT GCG CGC CCT CGG TG	GTA GTA GGA CAG GAA GTT GTT C
Cyclin E	CTC CAG GAA GAG GAA GGC AA	TCG ATT TTG GCC ATT TCT TCA
Cyclin A	GTC ACC ACA TAC TAT GGA CAT G	AAG TTT TCC TCT CAG CAC TGA C
p21	GTG ATT GCG ATG CGC TCA TG	TCT CTT GCA GAA GAC CAA TC
p27	GTC TAA CGG GAG CCC TAG CC	CTA ACC CCG TCT GGC TGT CC
p53	TGT GGA GTA TTT GGA TGA CA	GAA CAT GAG TTT TTT ATG GC
β-actin (reference gene)	TCA CCC TGA AGT ACC CCA TC	CCA TCT CTT GCT GCA AGT CC

Note: all primers are listed 5’-3’.

The software BIO-RAD CFX Manager (V 1.1.308) was used to relatively quantify the target genes according to the following equation [27]:

$$\text{Ratio}={\left({\text{E}}_{\text{target}}\right)}^{\Delta \text{Ct target}\left(\text{control}-\text{sample}\right)}/{\left({\text{E}}_{\text{ref}}\right)}^{\Delta \text{CtRef}\left(\text{control}–\text{sample}\right)}$$

In which (E): represents the amount of fold change per cycle per gene. Ref: represents the reference gene. Target: represents the target gene.

It has been well known that the ratio of Bax to Bcl-2 determines, in part, the susceptibility of cells to death signals [28]. Therefore, the Bax to Bcl-2 ratio was calculated using the following equation:

$$\begin{array}{l} \text{Bax/Bcl}-2\;\text{ratio}\\ \text{=}{\left(\text{mean PCR efficiency for Bax and Bcl}-2\right)}^{\left(\text{CtBcl}2–\text{CtBax}\right)} \end{array}$$

### Data analysis

All the data in the current study are shown as mean±2SE. The selectivity index (SI) was determined by using the ratio of CC50 to IC50. The data analysis was conducted by using SPSS software version (12.0.0.2). The effect of the tested extract on the inhibition of cell growth was evaluated by using 95% confidence intervals. IC50 and CC50 values were calculated using linear regression index equations. The statistically different effects of the extract on the ability of PBMC, HeLa and HepG2 cells to synthesize selected cytokines were compared with the control groups using the Student’s *t*-test. For flow cytomteric analysis, R2 fraction represented sub-G apoptotic cells; moreover, the percentage of cells at different cell cycle phases was calculated from the total cells minus apoptotic cells. For quantitative real time PCR, the up- or down- regulation of mRNA expression of selected genes was measured as expression fold changes in term of mean±2SD. The significance of up- or down- regulation of the normalized mRNA expression of selected genes was determined by comparing the mean ± 2SD of any up- or down- regulation with the mean ± 2SD of control (untreated cells), equal to 1 ± 2SD. P values less than 0.05 were considered significant.

## Results

### Cytotoxicity on human cancer cells

**Figure 1:**
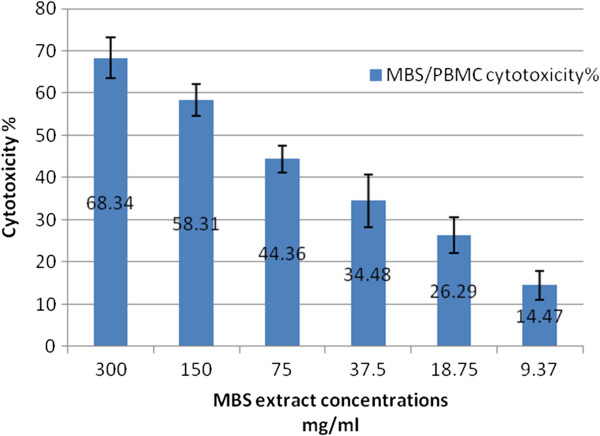
**The percentage of PBMC death after treatment with 2**-**fold serial dilutions of MBS extracts in term of mean **± **2SE** (**confidence interval CI 95%).**

**Figure 2:**
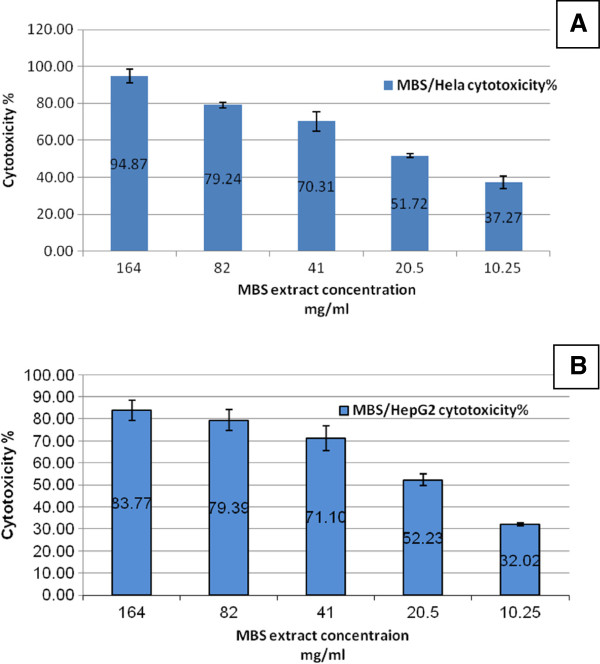
**The percentage of A**) **HeLa and B**) **HepG2 cells death after treatment with 2**-**fold serial dilutions of MBS extract in term of mean **± **2SE** (**confidence interval CI 95%.**

**Figure 3:**
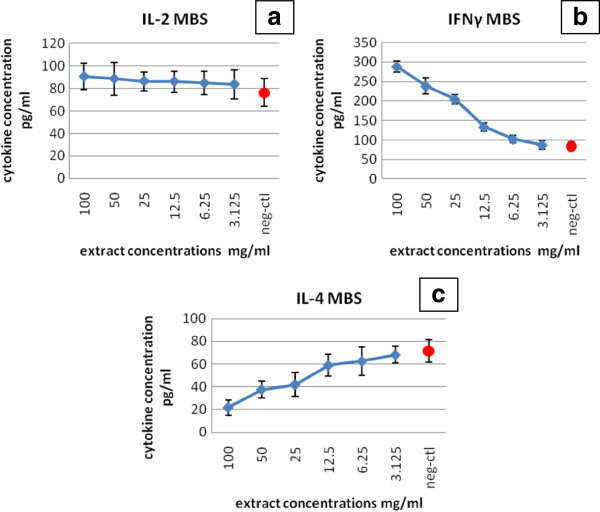
**Figures demonstrate the level of the produced cytokines,in comparison with negative control** (**neg**-**ctl**), **in culture supernatant of peripheral blood lymphocyte cell after treatment with mung bean sprout extract** (**MBS**): **a**) **IL**-**2**, **b**) **IFN**-**γ and c**) **IL**-**4.**

The results of the current study revealed that the cytotoxic effects of MBS extract on normal human cells (PBMC) was significantly different (P < 0.05) from that on human cancer cells (Table 2). The cytotoxic effect of MBS extract on PBMC, expressed as CC50, was 163.97 mg/ml while its IC50 on HeLa cells was 13.3 mg/ml and on HepG2 cells was 14.04 mg/ml. These findings revealed that MBS extract required high concentrations to be cytotoxic on normal human cells (Figure 1) while only low concentrations were enough to give the same effect on human cancer cells (Figure 2). These results reflected the good selectivity and safety of these extracts as cytotoxic agents (Figure 3).

**Table 2:** The cytotoxic effect of MBS extract on cancer cell lines

**Extract**	**Cancer cell line**	^**a**^**CC50 mg**/**ml**	^**b**^**IC50 mg**/**ml**	**P value**	**P** <**0**.**05**	^**c**^**SI**
MBS	HeLa	163.97 ± 5.73	13.3 ± 0.89	6.54539E-06	Significant	12.44 ± 0.83
	HepG2	163.97 ± 5.73	14.04 ± 1.5	7.25478E-06	Significant	11.94 ± 1.2

Note: ^a^The results of CC50 (on peripheral blood mononuclear cells) are shown as mean±2SE. ^b^The results of IC50 (on cancer cell lines) are shown as mean ± 2SE. ^c^Selectivity index represents the ratio of CC50/IC50. The results are shown as mean ± 2SE.

The cytotoxicity of MBS extract on PBMC, HeLa and HepG2 cells was dose dependent. In other words, the cytotoxicity of MBS extract decreased with higher dilutions, lower concentrations, of the extract. The significant differences of MBS cytotoxicity were supported by the results of the selectivity index (SI) which is the ratio of the highest concentration that causes 50% death to normal cells (CC50) to the lowest concentration that causes 50% death to cancer cells (IC50). The SI values of MBS extract demonstrated effective SI values on HeLa cells, 12.44, and HepG2 cells, 11.94 (Table 2). The cytotoxic effect of MBS extract showed no significant difference between HeLa and HepG2 cells.

### Non-specific immune response by PBMC

The results of the proliferation assay of PBMC treated with MBS extract were not significant. According to the formula of the proliferative %, the data of the proliferation % for PBMC were the same for that of the cytotoxicity % but in negative values (Figure 1). The results showed that MBS extract has no proliferative effect on PBMC when compared to the mitogenic effect of Con A (proliferative % = 63.89 ± 4.7). Instead, MBS extract showed a cytotoxic effect on these cells but this cytotoxic effect is of far less impact than that on the cancer cells.

Accordingly, PBMC were treated with extract concentrations less than that of the CC50 (Figure 3) to avoid any minute cytotoxic effect by the extract and to allow cytokines production, if any. The cytokine production assay showed that there was no significant difference (P > 0.05) in the level of IL-2 production between the treated PBMC and the control group, the untreated PBMC. Thus, PBMC treated with MBS extract did not produce IL-2 in a significant amount when compared with untreated PBMC (Table 3). However, there was a significant difference (P < 0.05) in the level of IFN-γ in PBMC culture supernatants of MBS-treated and MBS-untreated cells. It was shown that IFN-γ level in PBMC treated with MBS extract was high in cells treated with high concentrations of the extract; on the other hand, the level of IFN-γ decreased in dose dependent manner with decreasing concentrations of MBS extract indicating a stimulatory effect of MBS extract on the synthesis of IFN-γ by PBMC (Table 4). Moreover, the results indicated the dose dependent nature of IFN-γ synthesis by PBMC in response to MBS extract treatment.

**Table 3:** **The level of IL**-**2 produced by PBMC after treatment with different concentrations of MBS extract**

	**IL**-**2 concentrations**			
**Extract concentrations mg**/**ml**	*Treated cells pg/**ml**	*Negative control pg/**ml**	**P value**	**P** <**0**.**05**
100	90.6 ± 11.64	76.26 ± 12.3	0.41	Non-significant
50	88.47 ± 14.6		0.53	
25	86.42 ± 8.47		0.5	
12.5	86.11 ± 9.33		0.53	
6.25	84.75 ± 10.18		0.6	
3.12	83.63 ± 12.85		0.68	

Note: *All the results are shown as mean ± 2SE.

IL-2 level of each extract concentration was compared with that of the negative control (PBMC without extract).

**Table 4:** **The level of IFN**-**γ produced by PBMC after treatment with different concentrations of MBS extract**

	**IFN**-**γ concentrations**			
**Extract concentrations mg**/**ml**	*Treated cells pg/**ml**	*Negative control pg/**ml**	**P value**	**P** <**0**.**05**
100	288.63 ± 13.65	84.38 ± 11.5	< 0.0001	Significant
50	238.57 ± 20.5		< 0.0001	Significant
25	204.62 ± 12.64		< 0.0001	Non-significant
12.5	133.85 ± 10.53		0.006	Non-significant
6.25	102.49 ± 9.69		0.24	Non-significant
3.12	87.92 ± 11.22		0.83	Non-significant

Note: *All the results are shown as mean ± 2SE.

IFN-γ level of each extract concentration was compared with that of the negative control (PBMC without extract).

On the other hand, the level of Th2 cytokine, IL-4, in culture supernatants of PBMC treated with MBS extract was much lower than in untreated cells (P < 0.05). These findings demonstrated a significant decrease of IL-4 level, in dose dependent manner, with increasing concentrations of MBS extracts used in the treatment of PMBC. This reflects clearly an inhibitory effect of MBS extract on the production of IL-4 cytokine (Table 5).

**Table 5:** **The level of IL**-**4 produced by PBMC after treatment with different concentrations of MBS extract**

	**IL**-**4 concentrations**			
**Extract concentrations mg**/**ml**	*Treated cells pg/**ml**	*Negative control pg/**ml**	**P value**	**P** <**0**.**05**
100	21.63 ± 6.8	71.52 ± 9.94	0.001	Significant
50	37.5 ± 7.11		0.015	Significant
25	41.86 ± 10.4		0.022	Significant
12.5	58.9 ± 9.48		0.37	Non-significant
6.25	62.6 ± 12.56		0.62	Non-significant
3.12	68.19 ± 7.2		0.9	Non-significant

Note: *All the results are shown as mean ± 2SE.

IL-4 level of each extract concentration was compared with that of the negative control (PBMC without extract).

### Specific immune response by human cancer cells

**Figure 4:**
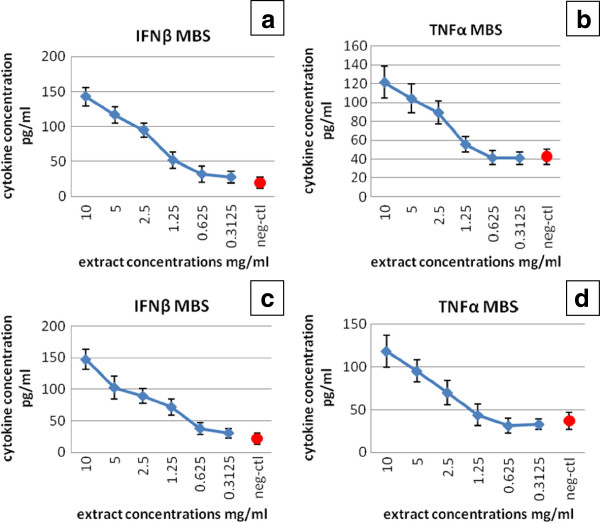
**Figures demonstrate the level of the produced cytokines**, **in comparison with negative control** (**neg**-**ctl**), **in culture supernatant of HeLa cell** (**a and b**) **and HepG2 cells** (**c and d**) **after treatment with MBS extract.**

The human cancer cell lines were treated with MBS extract concentrations less than the IC50 for each extract. These concentrations allowed the detection of the anticancer cytokines production in the culture supernatants of HeLa and HepG2 cells (Figure 4). The IFN-β levels in culture supernatants of HeLa and HepG2 treated with MBS extract showed significant and dose-dependent increase (P < 0.05) when compared to that of untreated cells (Tables 6 and 7). Accordingly, MBS extract revealed a clear stimulatory effect on the synthesis of IFN-β by both HeLa and HepG2 cells. Similarly, TNF-α levels showed a remarkable increase in the culture supernatants of HeLa and HepG2 cells treated with MBS extract when compared to that of untreated cells. Clearly, the MBS-driven increase of TNF-α levels was also in a dose dependent manner (Tables 8 and 9).

**Table 6:** **The level of IFN**-**β produced by cancer cells**, **HeLa**, **after treatment with different concentrations of MBS extract**

	**IFN**-**β concentrations**			
**Extract concentrations mg**/**ml**	*Treated cells pg/**ml**	*Negative control pg/**ml**	**P value**	**P** <**0**.**05**
10	142.85±13.18	19.54±8.23	<0.0001	Significant
5	116.83±11.52		<0.0001	Significant
2.5	94.8±10.32		<0.0001	Significant
1.25	51.67±11.47		0.04	Significant
0.625	31.67±11.3		0.4	Non-significant
0.31	27.31±8.47		0.73	Non-significant

Note: *All the results are shown as mean ± 2SE.

IFN-β level of each extract concentration was compared with that of the negative control (HeLa without extract).

**Table 7:** **The level of IFN**-**β produced by cancer cells**, **HepG2**, **after treatment with different concentrations of MBS extract**

	**IFN**-**β concentrations**			
**Extract concentrations mg**/**ml**	*Treated cells pg/**ml**	*Negative control pg/**ml**	**P value**	**P** <**0**.**05**
10	147.38±16.38	21.56±8.48	<0.0001	Significant
5	102.67±18.43		0.002	Significant
2.5	89.21±11.42		0.0001	Significant
1.25	71.68±12.73		0.006	Significant
0.625	37.5±9.58		0.23	Non-significant
0.31	30.17±7.34		0.6	Non-significant

Note: *All the results are shown as mean ± 2SE.

IFN-β level of each extract concentration was compared with that of the negative control (HepG2 without extract).

**Table 8:** **The level of TNF**-**α produced by cancer cells**, **HeLa**, **after treatment with different concentrations of MBS extract**

	**TNF**-**α concentrations**			
**Extract concentrations mg**/**ml**	*Treated cells pg/**ml**	*Negative control pg/**ml**	**P value**	**P** <**0**.**05**
10	121.72 ± 16.83	42.74 ± 8.26	0.001	Significant
5	104.36 ± 15.28		0.004	Significant
2.5	89.42 ± 12.16		0.007	Significant
1.25	55.69 ± 8.28		0.28	Non-significant
0.625	41.3 ± 7.39		0.89	Non-significant
0.31	41.13 ± 6.71		0.87	Non-significant

Note: *All the results are shown as mean ± 2SE.

TNF-α level of each extract concentration was compared with that of the negative control (HeLa without extract).

**Table 9:** **The level of TNF**-**α produced by cancer cells**, **HepG2**, **after treatment with different concentrations of MBS extract**

	**TNF**-**α concentrations**			
**Extract concentrations mg**/**ml**	*Treated cells pg/**ml**	*Negative control pg/**ml**	**P value**	**P** <**0**.**05**
10	118.47±18.3	37.21±9.98	0.002	Significant
5	95.28±12.89		0.003	Significant
2.5	70.14±14.22		0.08	Non-significant
1.25	43.85±12.63		0.68	Non-significant
0.625	31.56±8.91		0.67	Non-significant
0.31	33.16±6.38		0.74	Non-significant

Note: *All the results are shown as mean ± 2SE.

TNF-α level of each extract concentration was compared with that of the negative control (HepG2 without extract).

### MBS extract induced apoptosis and cell cycle arrest in human cancer cells

**Figure 5:**
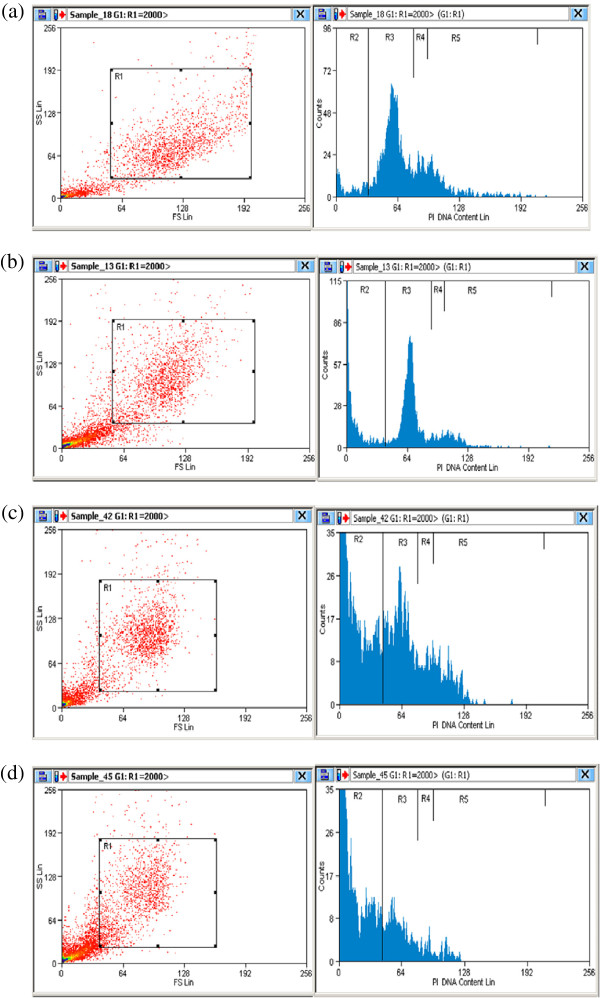
**DNA content frequency histograms representing HeLa cells after 24 h from** (**a**) **untreated cultures** (**b**) **cultures treated with MBS extract concentration** <**IC50** (**c**) **cultures treated with MBS extract IC50** (**d**) **cultures treated with MBS extract concentration** >**IC50.** The treatment affected the cell cycle distribution and induce apoptosis. The cells were stained with PI. Fluorescence of the PI-stained cells was measured using CyAn ADP appartus and Summit (V4.3) software. The software program provides the estimate of percentage of cells with fractional DNA content (apoptotic cells: R2) and cells in G0/G1 (R3), S (R4), and G2/M (R5) phases of the cycle. Total cell number (R1).

**Figure 6:**
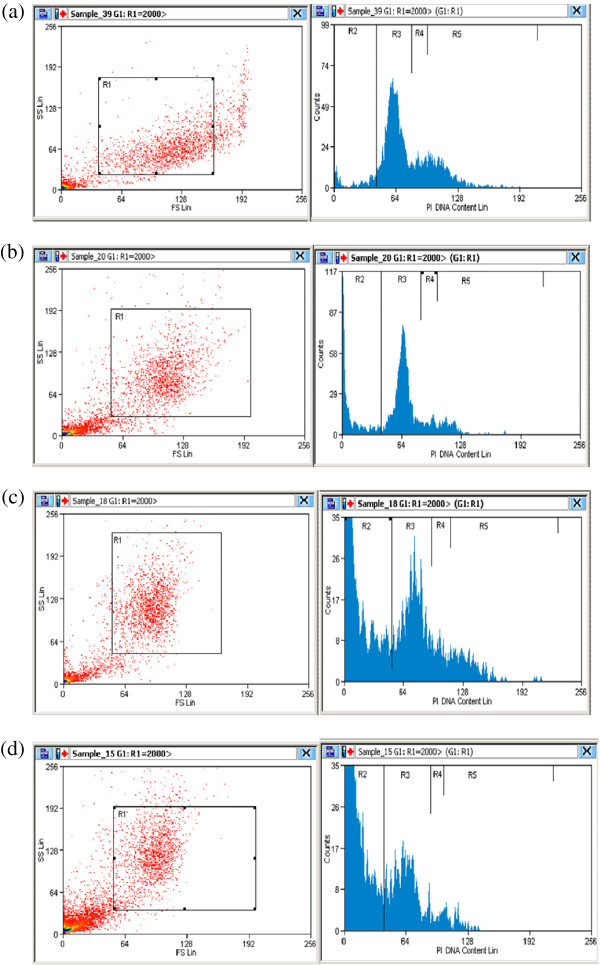
**DNA content frequency histograms representing HepG2 cells after 24 h from** (**a**) **untreated cultures** (**b**) **cultures treated with MBS extract concentration** <**IC50** (**c**) **cultures treated with MBS extract IC50** (**d**) **cultures treated with MBS extract concentration** >**IC50.** The treatment affected the cell cycle distribution and induce apoptosis. The cells were stained with PI. Fluorescence of the PI-stained cells was measured using CyAn ADP appartus and Summit (V4.3) software. The software program provide the estimate of percentage of cells with fractional DNA content (apoptotic cells: R2) and cells in G0/G1 (R3), S (R4), and G2/M (R5) phases of the cycle. Total cell number (R1).

**Figure 7:**
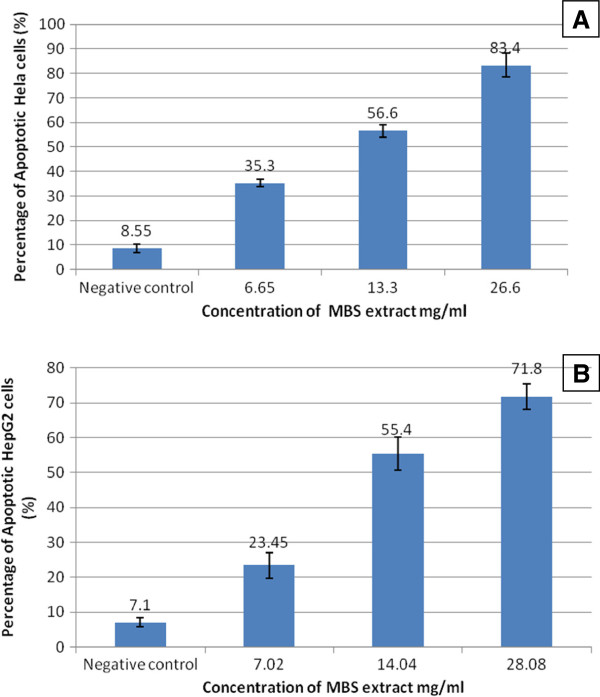
**A graph of flow cytometric analysis shows the percentage of apoptotic cells after treatment with MBS extract for 24 h in comparison with untreated cells** (**Negative control).** The extract’s concentrations represent 2-fold serial dilutions (concentration > IC50 > concentration). **A**) Apoptotic % of HeLa cells. **B**) Apoptotic % HepG2 cells. The increase in the percentage of the apoptotic cells was dose dependent.

The flow cytometric analysis showed possibility of MBS extract to induce apoptosis in the treated cells in comparison to untreated cells (Figures 5 and 6). By testing the apoptosis for fixed time interval, 24 h, and by using different doses of MBS extract, the percentage of the apoptotic cells were directly correlated with the concentration of MBS extract. MBS extract induced apoptosis, in a dose dependent manner, in treated HeLa and HepG2 cells while no observed apoptosis was found in untreated cells (P < 0.05). The IC50 of MBS extract induced apoptosis in 56.6 and 55.4% of HeLa and HepG2 cells, respectively, after 24 h of treatment (Figure 7).

**Figure 8:**
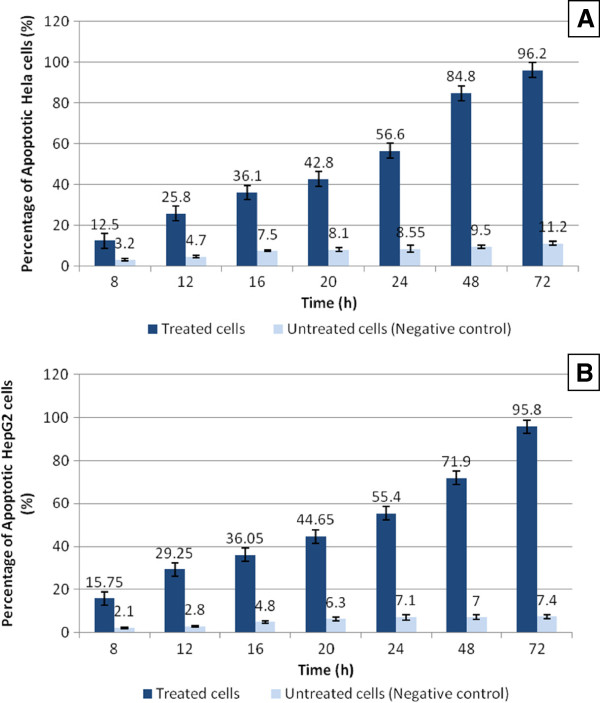
**A graph of flow cytometric analysis showes the percentage of apoptotic cells after treatment with MBS extract comparison with untreated cells.** The percentage of apoptotic cells increased with the time of treatment by extract IC50. **A**) Apoptotic % of HeLa cells. **B**) Apoptotic % of HepG2 cells. The increase in the percentage of the apoptotic cells was time dependent.

**Figure 9:**
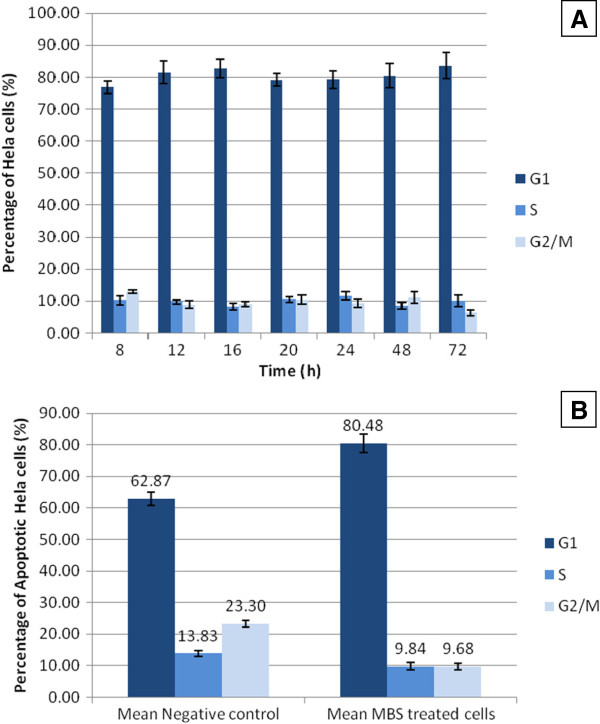
**A**) **Cell cycle arrest of HeLa cells treated by MBS extract IC50 at different time intervals. B**) The mean ± 2SE of the percentage of cells at G0/G1, S, and G2/M phases of the cell cylce of HeLa cells treated with MBS extract for different times in comparison with that of untreated cells.

**Figure 10:**
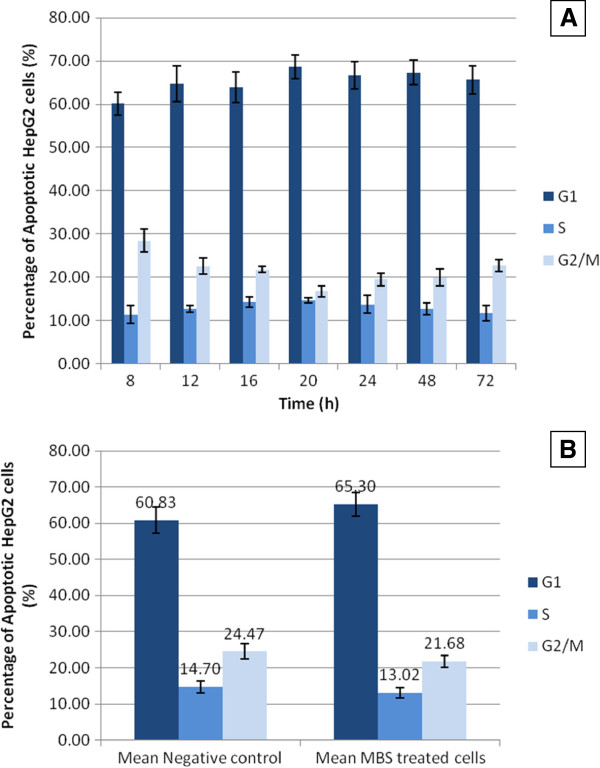
**A**) **Cell cycle arrest of HepG2 cells treated by MBS extract IC50 at different time intervals.B**) The mean ± 2SE of the percentage of cells at G0/G1, S, and G2/M phases of the cell cylce of HepG2 cells treated with MBS extract for different times in comparison with that of untreated cells.

By testing apoptosis at different time intervals, the flow cytometric analysis of HeLa and HepG2 cells treated with the IC50 of MBS extract showed that the extract provoked significant apoptosis (P < 0.05) in the treated HeLa and HepG2 cells in a time dependent manner when compared with untreated cells (Figure 8). There were no significant differences (P > 0.05) in the percentage of HeLa and HepG2 cells in different cell cycle phases when treated with MBS IC50 for different times (Figures 9A and 10A). However, MBS IC50 induced cell cycle arrest in G0/G1 phase in the treated HeLa, but not HepG2 when compared to untreated cells. The mean percentage of HeLa cells, treated with MBS extract for different times, in G0/G1 phase was higher than that in untreated cells (Figure 9B). The treatment with MBS IC50 increased the percentage of HeLa cells in G0/G1 phase from 62.87 ± 2.1%, in untreated cells, to 80.48 ± 2.97%. Alternatively, MBS IC50 did not increase significantly the percentage of HepG2 cells in G0/G1 phase from 60.83 ± 3.6, in untreated cells, to 65.30 ± 3.25% (Figure 10B).

### The apoptosis- and cell cycle- related genes in human cancer cells treated with MBS extract

**Figure 11:**
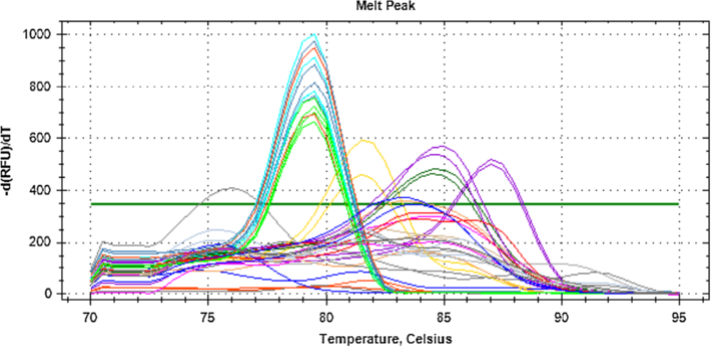
**The melting curve analysis of the PCR products at the end of the amplification was done by measuring the Eva Green fluorescence by slow heating at 0**.**5**°**Cs**^-**1**^**increments from 70 to 95**°**C**, **with continuous fluorescence collection.** Accordingly, a melting curve was generated at the end of the PCR amplification for monitoring the specificity of PCR reaction. It was found that a single peak (single product) at the expected melting temperature of PCR product, Tm 76-87°C, was observed while no significant premature peaks were found indicating that primer dimers artifacts or incorrect amplification products were minimal and providing further evidence on the specific detection of the target mRNA genes.

The results disclosed that 12, 16, and 20 h were the best times to study the expression level of the apoptosis- and cell cycle- related genes using real-time quantitative PCR. The other treatments of 8, 24, 48, and 72 h were ignored because they either gave very low or very high percentage of apoptotic cells. Studying the apoptosis- and cell cycle- related genes cannot be covered well during very early phase of extracts’ treatment during which not all apoptosis genes might be upregulated or downregulated; alike, during very late phase of apoptosis, most cells already died which renders measuring the expression of selected genes erroneous. A single peak at the expected melting temperature of PCR product, melting temperature (Tm) 76-87°C, was observed while no significant premature peaks were found indicating that primer dimers were minimal and providing further evidence on the specific detection of the target mRNA genes (Figure 11). The PCR efficiency of the primers used was greater than 90% and the correlation coefficients were greater than 0.99.

**Figure 12:**
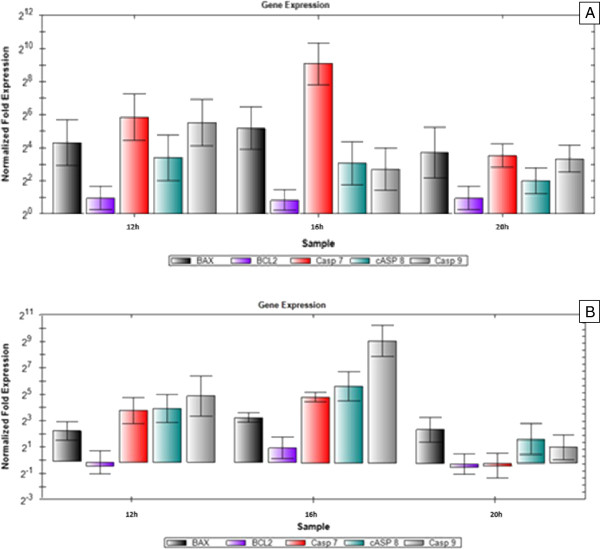
**Real**-**time quantitative PCR analysis illustrates the gene expression in** (**A**) **HeLa cells and** (**B**) **HepG2 cells after 12**, **16**, **and 20 h of treatment with the IC50 of MBS extract.** The gene expression was normalized with the reference gene (β-actin). The relative quantification of the target genes, Bax, Bcl-2, Caspase 7 (Casp 7), Caspase 8 (Casp 8), and Caspase 9 (Casp 9), by the delta–delta–C_t_ method was done using the software BIO-RAD CFX Manager (V 1.1.308).

**Figure 13:**
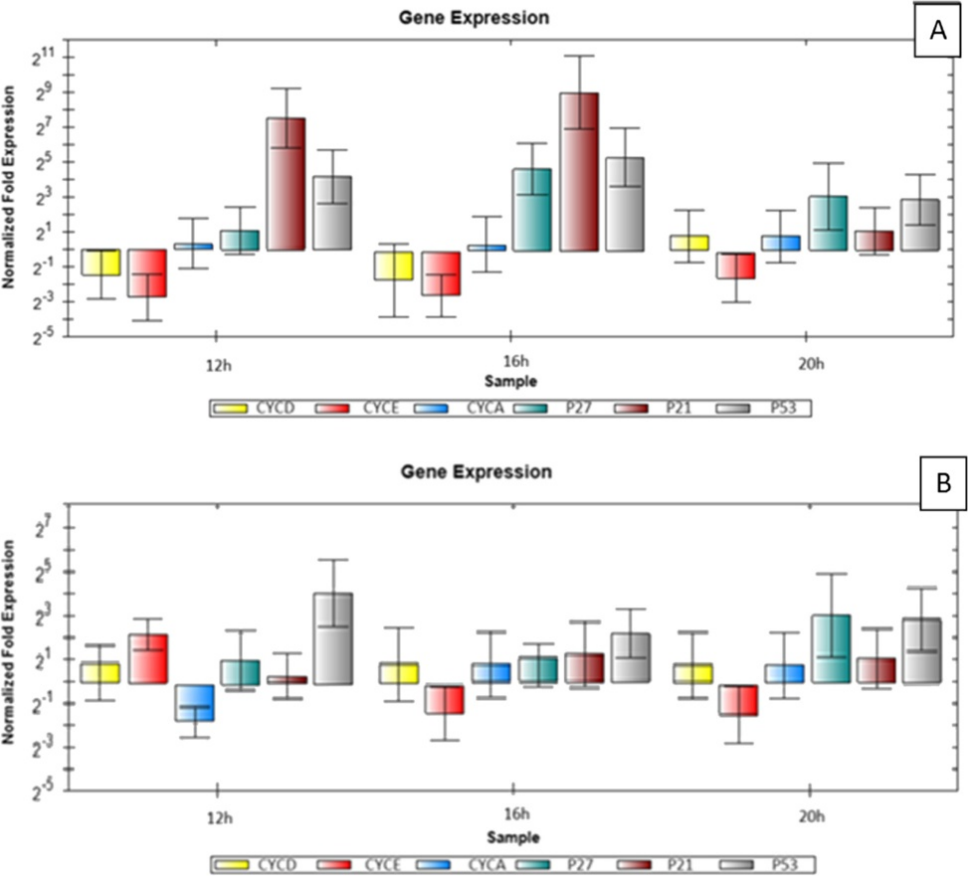
**Real**-**time quantitative PCR analysis illustrates the gene expression in** (**A**) **HeLa cells and** (**B**) **HepG2 cells after 12**, **16**, **and 20 h of treatment with the IC50 of MBS extract.** The gene expression was normalized with the reference gene (β-actin). The relative quantification of the target genes, Cyclin D (CYCD), Cyclin E (CYCE), Cyclin A (CYCA), p27, p21, and p53, by the delta–delta–C_t_ method was done using the software BIO-RAD CFX Manager (V 1.1.308).

MBS IC50 showed remarkable influence on the expression of the apoptosis-related genes in a positive and negative manner on both HeLa and HepG2 cells (Figures 12 and 13). MBS IC50 upregulated Bax gene expression in HeLa and HepG2 cells after 12, 16, and 20 h (P < 0.05). Bax upregulation at 16 h was not significantly different from that at 12 and 20 h (P > 0.05). Incubating HeLa and HepG2 cells with MBS IC50 did not show any effect on Bcl-2 gene expression after all the tested times of incubation (P > 0.05). MBS IC50 upregulated Caspase 7 gene expression after 12, 16, and 20 h of treatment of HeLa cells (P < 0.05). Alternatively, MBS IC50 upregulated Caspase 7 gene in the treated HepG2 cells only after 16 h (P < 0.05) with no significant difference from 12 h upregulation (P > 0.05). The expression of Caspase 8 gene in the treated HeLa cells was upregulated after 12, 16, and 20 h of treatment (P < 0.05) with no significant differences among the expressions of all of them (P > 0.05). At the same time, MBS IC50 upregulated Caspase 8 and 9 genes’ expressions in HepG2 cells only after 16h (P < 0.05) with no significant differences in their expressions from 12 h treatment (P > 0.05). On the contrary, MBS IC50 upregulated Caspase 9 gene expression in the treated HeLa cells after 12h (P < 0.05) with significant differences (P < 0.05) from that after 16 and 20h treatments.

The ratio of Bax to Bcl-2 proteins influences the apoptotic rate of cells; therefore, Bax/Bcl-2 ratio was calculated in treated and untreated HeLa and HepG2 cells (Table 10). The ratio of Bax/Bcl-2 in the treated HeLa and HepG2 cells after 12, 16, and 20h with MBS IC50 was higher than in the untreated cells (P < 0.05).

**Table 10:** **A table summarizes the susceptibilty of the treated and untreated HeLa and HepG2 cells to apoptosis depending on the ratio between the pro**-**apoptotic protein** (**Bax**) **and the anti**-**apoptotic protein** (**Bcl**-**2**)

**Extract**-**cells**	**Bax**/**Bcl**-**2 untreated**	**Bax**/**Bcl**-**2 treated 12h**	**P value**	**Bax**/**Bcl**-**2 treated 16h**	**P value**	**Bax**/**Bcl**-**2 treated 20h**	**P value**
MBS-Hela	0.31 ± 0.03	2.35 ± 0.22	0.004	10.71 ± 2.75	0.023	2.82 ± 0.55	0.016
MBS-HepG2	0.64 ± 0.06	2.75 ± 0.25	0.005	4.09 ± 1.01	0.027	4.22 ± 1.25	0.038

Note: the ratio was calculated as mean ± 2SE.

The ratio of the treated cells was calculated after 12, 16, and 20 h of treatment with the IC50 of MBS extracts.

Because flow cytometric analysis showed cell cycle arrest by MBS extracts at G0/G1 phase, the regulatory proteins of G0/G1 phase in the mammalian cell cycle, cyclin D, E, and A were studied. These cyclins are responsible for the activation of cyclin-dependent kinases (cdk) in G1 and S phases of the cell cycle of HeLa and HepG2 cells. Moreover, the mRNA expression of the proteins responsible for the inhibition of cyclin-cdk active complexes of the G1 and S phases of HeLa and HepG2 cells exposed to the IC50 of MBS extracts were studied as well, namely tumor suppressor proteins p27, p21, and p53 (Figure 4.24 and Figure 4.25).

The current findings of real-time quantitative PCR were congruous with that of flow cytometry. In addition, the results of real time PCR granted valuable details on some aspects and mechanisms that underlie the cell cycle arrest ability of MBS extract. For MBS extract effect on HeLa cells, it was found that the expression of both cyclin D and E, cyclins of G0/G1 phase, was downregulated after 12, 16, and 20h exposure of HeLa cells to MBS extract with no significant differences (P > 0.05) while the expression of cyclin A, cyclin of late G1 and S phase, was not affected by MBS extract (P > 0.05). However, the cdk-inhibitory protein, p27 was significantly upregulated after 16 h (P < 0.05) while its upregulation after 12 and 20h was not significant (P > 0.05) indicating that p27 peak of upregulation was around 16h. For p21 and p53, both of them were largely upregulated, especially p21, after 12 and 16 h (P < 0.05) but not after 20 h (P > 0.05). The upregulation of p21 reached 128 folds of expression after 12 h and above 512 folds of expression after 16 h while the upregulation of p53 reached 16 folds after 12 h and more than 32 after 16 h. Both p53 and p21 are expressed together for the inhibition of cyclin-cdk complexes and peaked after 16 h of cells exposure to MBS extract indicating a clear inhibiting role of MBS extract for the cell cycle at G0/G1 through cdk-inhibitory proteins rather than affecting much the expression level of cyclins themselves.

The effect of MBS extract on HepG2 cells was somehow different from its effect on HeLa cells. MBS extract did not show, via both flow cytometry and real time PCR, a remarkable inhibitory effect on the cell cycle of HepG2 cells. The mRNA expression level of cyclin D and A was not affected by MBS extract (P > 0.05) while cyclin E was upregulated after 12 h (P < 0.05). Moreover, the expression of p27 and p21 proteins did not reach the significant level of upregulation (P > 0.05) indicating that MBS extract has little inhibitory effect on the cell cycle of HepG2 cells. Nevertheless, p53 was the only protein shown to be significantly upregulated after 12 and 20 h (P < 0.05) and borderline upregulation (P = 0.048) after 16h. However, it is not well understood why p53 was upregulated while other tumor suppressor proteins were not. Collectively, the current results of the effect of MBS extract on HepG2 are in harmony with flow cytometry results. Both assays revealed weak inhibitory effect of MBS extract on HepG2, but not HeLa, cells indicating a cell-specific activity of MBS extract on the cell cycle of different types of cells. The sole upregulation of p53 by MBS extract explains some aspects of the remarkable apoptotic activity of MBS extract towards HepG2 cells.

## Discussion

The cytotoxic effects of MBS extract was investigated on two of the most important types of cancer in Asian countries. Depending on recent studies, hepatocellular carcinoma, a liver cancer, is considered to be one of the most common cancers worldwide with an extremely poor prognosis. Moreover, it ranks as the second leading cause of cancer-related deaths in China and many Asian regions [29]. On the other hand, cervical cancer continues to be the commonest cause of death among women in developing countries. In addition, it is the second most frequent cancer among females worldwide [30].

The findings of the current study revealed effective cytotoxic effects on HeLa and HepG2 cells by MBS extract. No previous studies have investigated the cytotoxic effect of MBS extract. Interestingly, in the current study, MBS extract showed selective cytotoxic effects against both HeLa and HepG2 cells with SI values of 12.44 and 11.94, respectively. Taken into account the SI biological efficacy, or SI, ≥ 10 is considered not due to non-specific cytotoxicity, the SI values of MBS extract reflect remarkable selectivity. There is a need to find new chemical agents able to differentiate between normal and cancerous cells. This is a necessary criterion to selectively kill cancer cells and this such selective natural proidcts have become highly needed [31]. It has been proven that germination of the mung bean causes a rise in the total content of the antioxidant components like phenolic compounds, α-tocopherol and vitamin C [32]. The absence of significant differences in MBS cytotoxic effects on HeLa and HepG2 cells may highlight the common cytotoxic mechanisms that are possibly used by the extract to seize the cell growth of both cell lines. One of the possible mechanisms responsible for the cytotoxic effect of MBS extract is the great possibility of flavonoids and phenolics in reducing alkylperoxyl radical (ROO·) content which has a role in radical-mediated pathogenesis such as carcinogenesis. It was found that the scavengers of alkylperoxyl radical (ROO·) may play an important role in cancer prevention [33]. Another possible mechanism that may explain the results of this study is the synergistic effect of phenolic compounds and α-tocopherol. It was observed that phenolic compounds indirectly increase the (ROO·)-scavenging capacity in vivo by increasing the level of α-tocopherol [33]. It is known that α-tocopherol, one of vitamin E family members, has a potent antioxidant activity [34]. In addition, many studies proved the efficacy of α-tocopherol as antithrombotic, anticoagulant, neuroprotective, antiproliferative, immunomodulatory, cell membrane-stabilizing, and antiviral [35-38]. Thus, the clear cytotoxic effect of MBS extract may be related to the efficacy of α-tocopherol alone [39] or, more likely, together with phenolic compounds. Besides, the presence of anticarcinogenic substances, such as vitamin C, [40] in the germinated mung bean sprouts may enhance the cytotoxic effect of MBS extract.

MBS extract was shown to be a good inducer for both anticancer cytokines, i.e., TNF-α and IFN-β in culture supernatants of HeLa and HepG2 cells. There was a significant increase in their levels in MBS-treated cells when compared to untreated cells. The increase was dose dependent which reflected the capability of MBS extract as an inducer to the production of these two essential anticancer cytokines. Both TNF-α and IFN-β are important cytokines to regulate cell growth and death [41]. A recent study found that TNF-α and IFN-β are major inducible cytokines that function to counteract cellular transformation in a synergistic action [42]. Accordingly, the current study clarified that MBS extract induced the production of TNF-α and IFN-β from human cancer cells, and this led to the inhibition of the growth of these cells and this might lead to the death of treated human cancer cells. The cytokines synthesized by cancer cells are released to culture medium and then bind to their receptors on the cell surface of cancer cells, leading to cell growth arrest and apoptosis via an autocrine pathway [22]. The anticancer activity of IFN-β has been well recognized [43], whereas TNF-α plays a paradoxical role in carcinogenesis [44]. The ability of MBS extract to induce the production of anticancer cytokines was in agreement with some recent findings. Several studies demonstrated that α-tocopherol is considered as a potent antitumor agent which increases apoptosis and decreases proliferation in tumor cells [45,46]. Similarly, vitamin C proved to have cytotoxic action on human cancer cells and induce apoptosis [47]. And, it was found that vitamin C is capable to induce TNF-α production *in vivo*[48]. Thus, the cytotoxic effect of MBS extract could be partly explained by the induction of anticancer cytokines which in turn induces cell death; moreover, all these actions may be induced by the two important components of MBS extract, i.e., α-tocopherol and vitamin C.

Besides inducing anticancer cytokines, there was a need to explore the pro-apoptotoic effect of MBS extract on cancer cells. The flow cytometry analysis was done to investigate the presence or absence of the apoptotic cells in the treated HeLa and HepG2 cells. The results revealed that MBS extract induced apoptosis in the treated cells in a dose and time dependent manner with significant differences from untreated cells. The cell cycle arrest and the induction of apoptosis in cancer cells have become major indicators of anticancer effects [49]. Moreover, the antitumor effects could be attributed to altered biochemical mechanisms, including inhibitions of proliferation, induction of cell cycle arrest at various cell cycle checkpoints, and enhanced apoptosis [50]. The current study revealed that treated HeLa and HepG2 cells with the IC50 of MBS extract induced cell cycle arrest significantly in G0/G1 phase in HeLa but not in HepG2 cells. The mean percentage of HeLa cells, treated with MBS extract for different times, in G0/G1 phase was higher than in untreated cells (Figure 9). The treatment with MBS IC50 increased the percentage of HeLa cells in G0/G1 phase from 62.87 ± 2.1%, in untreated cells, to 80.48 ± 2.97%. Alternatively, MBS IC50 did not increase significantly the percentage of HepG2 cells in G0/G1 phase from 60.83 ± 3.6, in untreated cells, to 65.30 ± 3.25% (Figure 10). The results of this study were in agreement with other previous studies which found that flavonoids, phenolic acids, and other antioxidants inhibit the cancer cell cycle progression, cell proliferation and tumor growth. In addition, they can prevent tumor metastasis by inducing cell-cycle arrest and apoptosis [51,52].

Subsequently, real-time quantitative PCR was used to confirm the deteted aoptotisis by flow cytometry and to detect the underlying mechanism(s) used by MBS extract to induce apoptosis. To demonstrate these mechanism(s), the expression of several apoptosis-related genes was investigated in the current study. Apoptosis is a broad network of signals that act through two major apoptotic pathways: the extrinsic death receptor pathway (via caspase 8), which triggers the activation of a caspase cascade, and the intrinsic mitochondrial pathway (via caspase 9), which shifts the balance in the Bcl-2 family towards the pro-apoptotic members and, consequently, toward caspase-mediated apoptosis [53]. Both caspase 8 and 9 are considered as initiator caspases which in turn can activate the effector caspases, namely caspase 3 and caspase 7, leading to dramatic morphologic changes of apoptosis [54]. In the current study, MBS extract was found to be a potent inducer to the extrinsic pathway of apoptosis via caspase 8. MBS extract upregulated the gene expression of caspase 8 after 12 h of treatment in both HeLa and HepG2 cells. Caspase 8 upregulation continued till 20 h in the treated HeLa cells while it continued maximally till 16 h in the treated HepG2 cells. It has been shown that the activation of caspase 8 requires the involvement of apoptotic ligands such as TNF-α and Fas ligand [53]. As mentioned earlier, MBS extract stimulated TNF-α production in the culture supernatants of the treated HeLa and HepG2 cells. Thus, we propose that TNF-α which was produced by the treated cells might have an autocrine effect on the same producing cells. And it activated the extrinsic apoptosis pathway via caspase 8 by binding to its receptors on the surface of cancer cells.

The current results revealed that MBS extract upregulated the expression of Bax gene after 12 h of treatment and this upregulation continued till 20 h. It was stated that cytochrome *c* release from mitochondria could be controlled by Bax. And the translocation of Bax can alter the outer mitochondrial membrane permeability, leading to cytochrome *c* release from the mitochondria to the cytosol then activation of the intrinsic apoptosis pathway [55]. Accordingly, MBS extract promoted the intrinsic apoptosis pathway by its ability to upregulate Bax gene. Moreover, the results demonstrated the predominance of Bax gene over Bcl-2 gene in all of the treated cells. It was proven that the ratio of Bax to Bcl-2 determines, in part, the susceptibility of cells to death signals [28]. For that reason, Bcl-2 proteins have emerged as an attractive target for the development of novel anticancer drugs, and this could be one of the targets hit by MBS active compounds to induce apoptosis. Although, there was no effect on the level of Bcl-2 expression in the treated HeLa and HepG2 cells with MBS extract, the Bax/Bcl-2 ratio in cells treated with MBS extract was high after 16h, for HeLa cells, and was high after 20h, for HepG2. Hence, this ratio may explain in part the susceptibility of the MBS-treated HeLa and HepG2 cells to apoptosis.

The upregulation of caspase 9 in HeLa and HepG2 cells was clear after 12h of treatment with MBS extract. It is well known that caspase 9 can be activated by caspase 8 or can be activated independently on binding of cytochrome *c* release from the mitochondria [56]. We assumed that the intrinsic apoptosis pathway induced by MBS extract in the treated cancer cells might be provoked via direct upregulation of caspase 9 gene, via the activation of caspase 8 by the extrinsic pathway, or via the upregulation of Bax gene. In addition, MBS extract induced the expression of caspase 7 gene in the treated HeLa and HepG2 cells after 12h. Caspase 7 can be activated by both extrinsic and intrinsic pathways of apoptosis. Moreover, caspase 7-dependent pathway without caspase-3 activation is recently considered as caspase-independent apoptosis pathway. And caspase 7 can activate caspase 12 which results in the induction of apoptosis during endoplasmic reticulum stress [57]. Therefore, the results of the current study disclosed a fact that MBS extract might induce apoptosis by different pathways. The reason behind these results is that we are dealing with a curde extract with a large number of different components that could trigger different pathways of apoptosis. Accordingly, using MBS extract might have advantage orver single active componnents in triggering two or three pathways of apoptosis simultaneously leading to vigorous induction of apoptosis. The induction of multi-pathway apoptosis usually leads to effective anticancer activity able to overcome any resistance that might issue from cancer cells against apoptotic signals or against one of the apopotitic pathways. For this reason, finding new natural anticancer products has increasingly become a favorable trend of treating cancer. In addition, the current results highlight the ability to isolate more than one effective cytotoxic component from MBS extract.

The regulatory proteins of cell cycle evaluated in the current study were chosen carefully in order to give further image on the underlying mechanisms for the observed G0/G1 arrest as well as apoptosis found in MBS-treated HeLa and HpeG2 cells. The studied markers were the cdk-activating proteins, namely cyclins and cdk-inhibitors, namely CKI or tumor suppressor proteins, such as p27, p21, and p53. Interestingly, MBS extract succeeded in inducing all the studied cdk-inhibitors, p21, p53, and p27 in HeLa cells while it induced only p53 in HepG2 cells. This is a clue for the cell type- specific interaction of MBS extract. This feature necessitates studying the cell growth- inhibiting activity of plants’ extracts individually on different human cancer cell lines. The peak time for the induction of tumor suppressor proteins was after 16 h. Therefore, after 20 h, most affected cells died due to either cell cycle arrest or apoptosis. Upon comparing the current results with these of flow cytometry, it is obvious that both results are in harmony. Via flow cytometry, MBS extract caused slight and insignificant arrest in G1 phase of cell cycle of HepG2 cells but not HeLa cells. This feature was explained by the results of real time PCR. MBS extract showed weak inducing capability to cdk-inhibitors, p21, p53, and p27 in HepG2 cells while it largely induced p21, p53, and p27 in HeLa cells.

The current findings of the cell cycle inhibitory effects of MBS extract showed weak or absent influence on the expression of cdk-activating cyclins. Instead, MBS extract showed remarkable induction and upsurge of cdk-inhibitor proteins. Therefore, it is concluded that these cdk-inhibitor proteins are the main mechanism pursued by MBS extract to exert the G1 cell cycle arrest and ultimately the final fate, death of cells.

In addition, the current study revealed an interesting finding on MBS extract; it induced synthesis of TNF-α from cancerous cells. TNF-α can be the central link between the extract and its remarkable ability to induce cdk-inhibitors and/or to downregulate cyclins. TNF-α was found to induce p21 (waf1) protein in tumor cells, and it also induces p21 binding to CDK 2/4 and 6 complexes resulting in the inhibition of their activities [58]. This inhibition drives cells to G1 arrest. In addition, p27Kip1 was reported to induce caspase -dependent and -independent phases of cell death through TNF-α signaling [59].

In the current study, another antitumor cytokine was found to be secreted by tumor cells in response to MBS extract, namely IFN-β. Like TNF-α, IFN-β is most probably linked to the apoptotic and cell cycle slowing/arresting potential of MBS. Interferons inhibit the growth of tumor cells by blocking the progression of their cell cycle via the upregulation of the cyclin-dependent kinase inhibitor p21 (waf1), [60]. Moreover, TNF and IFN molecules were shown to synergistically induce a G1 arrest associated with reduced levels of cyclin D1 and cdk2, and increased expression of the cdk inhibitors p16INK4a, p21WAF1 and p27Kip1 [61]. In a recent study, IFN-β signaling was shown to repress telomerase activity in ovarian cancer and this signaling was found to be mediated by p21(waf1) [62]. Interestingly, two previous studies proved the positive signaling pathway between IFN-β and p53 and p21 proteins in inducing cell cycle arrest and apoptosis [63,64].

### Immunomodulatory activity of MBS extract

The reason behind studying the immunomodulatory effect of MBS extract is that MBS is rich in flavonoids [65]. These compounds are able to stimulate CD4^+^ T lymphocytes that represent the major source of the IL-2 cytokine [66]. However, this study revealed a non-significant effect of MBS extract on IL-2 production from human PBMC. These results were supported by the findings of the proliferation assay which was performed on the same cells treated with MBS extract. The results showed negative effect of MBS extract on PBMC proliferation instead of a positive effect.

Cell-mediated immune response is an important aspect of host resistance to infection and cancer. It is thought to be tightly regulated by balance between type 1 cytokines (Th1) including IL-2, IFN-γ, TNF-α, and IL-12 and the type 2 cytokines (Th2) such as IL-4, IL-6, and IL-10 [13]. Immunomodulators can be divided into main three groups, i.e., immunostimulating, immunosuppressive, and immunopolarizing agents, which all are useful for different therapeutic needs [67]. MBS extract was found to lack the immunostimulatory effect. Nevertheless, MBS extract was shown to shift the polarization of PBMC towards type 1 (Th1) rather than type 2 (Th2); this polarization determines the prognosis of many infectious diseases. And most importantly MBS-polarization can shift immune response from humoral to cell-mediated immunity (CMI) where the anti-tumor immune cytotoxicity lies. The immunomodulatory effect of MBS extract was evaluated by studying the production of IFN-γ and IL-4 by PBMC cultured *in vitro* with MBS extract. IFN-γ and IL-4 are key cytokines for the development of type 1 and type 2 immune responses, respectively [68]. The current study showed that MBS extract increased reamrakably IFN-γ and decreased IL-4 levels in the supernatant of PBMC culture when compared with untreated (control) cells. The increase and the decrease in IFN-γ and IL-4 levels, respectively, were dose-dependent; the highest increase in IFN-γ or the highest decrease in IL-4 levels were driven by the highest concentration used of MBS extract, 100 mg/ml. The immunopolarizing effect of MBS extract towards Th1 immune response could be in agreement with a previous study which found that α-tocopherol induces high secretion of IFN-γ *in vivo*[69]. The role of IFN-γ in enhancing antitumor immunity has been well proven by inducing CD8^+^ cells-based cellular cytotoxicity and by inducing abundant production of IL-12 which stimulates natural killer (NK) cells that act together with CD8^+^ cells as the main immunological cytotoxicity defense line against tumors [70]. Moreover, ascorbic acid (vitamin C), the other major components of MBS extract, also induces IFN-γ production by stimulating Th1 pathway cytokines [71,72]. In addition, the immunopolarizing effect of MBS extract shown in this study could be supported by a previous study which found a remarkable hepatoprotective role of mung bean aqueous extract in curing liver injury during hepatotoxicity [73]. The hypothesis behind the hepatoprotective action of mung bean aqueous extract could be due to its ability to induce the production of IFN-γ. Interferon gamma is produced by certain subsets of natural killer T cells in the liver which are the primary mediators of antitumor responses [74,75]. Accordingly, in addition to the direct antitumor cytotoxicity and the induction of antitumor cytokines by MBS extract, the third antitumor mechanism of MBS could be attributed to the induction of IFN-γ production and immunoplorization of immune resposne towards CMI and cellular cytotoxicity.

## Conclusion

The cytotoxic effects of MBS extract were investigated thoroughly. The current study showed significant cytotoxic effects exerted by MBS extract against human cervical cancer cells and human hepatocarcinoma cells. The cytotoxicity of MBS extract to HeLa and HepG2 cells was shown to be hgihly selective. Moreover, MBS extract was found to act as a potent inducer for apoptosis in the treated human cancer cells via caspase-dependent, both extrinsic and intrinsic pathways, and may be caspase-independent pathway. MBS extract induced apoptosis and cell cycle arrest in the treated human cancer cells via cell-type specific interactions. Cdk-inhibitor proteins (p21, p27, and p53) were the main mechanisms used by MBS extract to exert G1 cell cycle arrest and ultimately the final fate of cells, apoptosis.

In addition, MBS extract induced synthesis of TNF-α and IFN-β from cancerous cells and this might be associated with the apoptotic and cell cycle slowing/arresting capability of MBS extract. Interestingly, MBS extract was shown to be an immunopolarizing agent by inducing IFN-γ and inhibiting IL-4 production by PBMC. This triggers CMI and cellular cytotoxicity which are critical defense mechanisms against cancer, allergy, and different pathogens.

Taken together, the findings of the current study indicated that MBS extract is a highly promising cytotoxic agent by affecting either known or new targets in the anticancer chemotherapy. And these anticancer activities were thought to be driven by more than one component giving chance for MBS extract to exert strong, multi-mechanism, and synergistic anticancer and/or immunomodulatory effects. As the current study is unpredecented in exploring the anticancer activitieis and underlying mechanisms of MBS extract, further study is needed urgently to identify the bioactive compounds responsible for the discovered anticancer and immonumodaltory powerful and selective activities of MBS extract.

## Competing interests

Authors declare that there are no competing interests associated with the current study.

## Authors’ contributions

RRH and ASA contributed equally to this work; RRH and ASA designed the work; FAJ provided vital reagents and analytical tools; ASA and FAB involved in editing the manuscript; ASA, ZS, and FA analyzed the data; FAB provided the financial support for this work; RRH wrote the manuscript. All authors have read and approved the final manuscript.

## Pre-publication history

The pre-publication history for this paper can be accessed here:

http://www.biomedcentral.com/1472-6882/12/208/prepub
